# Calcium Sulphydrate; a Safe, Efficient and Economical Depilatory*By Dr. A. W. Brayton, of Indianapolis, Editor *Indiana Medical Journal*; Professor of Dermatology and Syphilology in the Medical College of Indiana; Dermatologist to the Indianapolis City Hospital and City Dispensary.

**Published:** 1897-03

**Authors:** 


### CALCIUM SULPHYDRATE; A SAFE, EFFICIENT, AND ECONOMICAL DEPILATORY.*

On the 720th page, Vol. I., of *Watts' Dictionary of Chemistry* (London, Longman & Green, 1872), this paste is spoken of as follows: "Sulphydrate of calcium may be used as a depilatory, and is recommended for this use by Boettger (Am. Chem. and Phar. xxix. 79) in place of sulphide of arsenic. It may be prepared by passing sulphuretted hydrogen into thin milk of lime till the mass assumes a bluish-gray color (from admixed sulphide of iron). The paste thus formed is laid to the thickness of a line on the surface from which the hair is to be removed, and scraped off after a minute or two, with a blunt knife, the hair then coming away with it. If it could be produced cheaply enough it might be well used for removing the hair from hides in the tan-yard."

The only reference I find to this paste in any work on dermatology is on the 448th page of *Kaposi's Diseases of the Skin*, (Wm. Wood & Co., New York, 1895) as follows: "Sulphide of calcium paste which is prepared by the introduction of sulphuretted hydrogen into hydrated lime (Boettger's paste), acts still more rapidly than the Oriental paste, and is therefore more convenient in practice, especially as it can be procured ready-made from the druggist."

Calcium sulphydrate ($CaH_2\ S_2$) is best prepared by passing sulphuretted hydrogen gas through a thick cream of well slacked lime until the lime will absorb no more of the gas. It requires about two hours to make a pint. The apparatus should be set up in a fume chamber or else in the open air.

The solution thus prepared has a sharp, bitter taste, an alkaline reaction, and is moderately caustic. The chemical change is expressed as follows: $CaH_2O_2+2(H_2S)=CaH_2S_2+2(H_2O)$.

Kaposi states that pastes are better than razors for the palliative treatment of disfiguring hypertrichosis. He recommends the caustic Oriental paste, known in the Eastern harems as "rusma" and used to destroy the pubic and axillary hair. It is made of yellow sulphide of arsenic, one-half ounce; unslacked lime, eight ounces; mixed with water to a paste and

---

*By Dr. A. W. Brayton, of Indianapolis, Editor *Indiana Medical Journal*; Professor of Dermatology and Syphilology in the Medical College of Indiana; Dermatologist to the Indianapolis City Hospital and City Dispensary.

boiled. It is put on by means of a spatula and left ten minutes till it dries and then scraped off with a dull knife. The skin is then washed, dried and powdered. This is probably the oldest and best-known Eastern depilatory. It has been used for ages among the Jews "for the periodical removal of the stubble of the beard." The fact that it contains an arsenical compound seems to debar Western people from its use.

No one can deny that efficient depilatories are to be had on the market. But excessive prices are asked for them, and they are sold as secret remedies. Nothing is more certain, also, than the fact that the ordinary depilatories, as put up by our druggists and described in our skin books—for example, those given by Anderson (barium sulphide) and by Dr. Duhring (sodium sulphide)—do not remove the hair. The authors do not speak of them as though they were in common use in their practice, but simply quote the prescriptions from one another. Finding that these prescriptions, as put up by druggists, failed, I asked Ansel Moffett, a well-known chemist of Indianapolis, as to the reason of their failure. He referred me to Boettger's paste as a perfect depilatory, and one easily and cheaply made. A more careful reading of Kaposi by one of my students revealed the brief mention of it in Kaposi's new work above referred to. Mr. Moffett made a pint of the green paste, which was active, pleasant and safe to use, and was at once applied to the entire hairy arms of several students before the public clinic at the City Hospital and before the senior class of the Indiana Medical College.

It was the thickness of pancake batter, and was kept in a salt-mouth pint bottle, with the stopper well smeared with vaseline. It still retains its perfect depilatory power at the present writing (May 27th), after having been made six months.

Dr. W. N. Wishard has used it in removing the pubic and scrotal hair preceding operation. Dr. Jos. Marsee has also used it, and approves it.

I believe such a depilatory as sulphydrate of calcium paste has a wide field in genito-urinary and abdominal surgery. It is antiseptic and alkaline; it is, therefore, an efficient soap and bleaching agent; the skin after its use is much whitened by the removal of dirt and superficial epithelium and oil, as well as by the removal of the hair. I have found that the hairs are destroyed some distance down in the mouth of the follicle; much closer than by shaving. There is no more folliculitis incited than by ordinary close shaving. Any close scrubbing of the skin with soap followed by shaving will open up the follicles, particularly on the back of the neck, to the entrance of cocci and may be followed by a mild folliculitis or even furunculosis. A dusting powder of stearte of zinc may be advantageously used after the depilatory has been washed off in abundant water and the skin well dried. Mr. Moffett shaved himself with this depilatory with success, and suffered no after inflammation; it required five minutes to destroy the stronger stubble at the point of the chin.

I have applied the paste directly with my fingers and palms, as one would do in washing the arms with soap. But after several times using it in this way experimentally on several persons, I found the skin on the tips of the fingers and the ends of the nails were softened and tender. A swab would be a safer method of applying it where one uses it frequently.

I presented the method of using Boettger's paste to the dermatological section at the Atlanta meeting of the American Medical Association, recommending its use in ringworm of the scalp. The hair can be cut closely, the paste applied, washed off, and followed by the selected parasiticide.

Dr. Bulkley, the chairman of the skin section, was much interested in this mode of treatment, and so also were others who asked for the formula, the mode of preparation, and requested me to publish my observations on its use.

As to the cosmetic uses of such a paste, these I leave to the profession. For hypertrichosis of the chin and lip, scattering hairs, moles, etc., I use only the negative needle of a small galvanic battery, according to the method of Hardaway. But if one has a patient suffering with hypertrichosis, the use of this paste once a week or two weeks will be safe, economical and efficient. I am sure also that it has a wide application in surgery, and in the treatment of the various ringworms. It may be used to cleanse the hands and arms of the abdominal surgeon of hair and deep-seated filth in the follicles, from the finger-tips to the elbows, before laparotomy. The hair will grow again and require removal in from two to three weeks.

*24½ West Ohio Street.*

